# Funding Medicaid codes for screening and brief intervention services: lessons learned

**DOI:** 10.1186/1940-0640-8-S1-A2

**Published:** 2013-09-04

**Authors:** Rita E Adkins, Joseph G Grailer, Mandy R Lay, Barbara E Keehn

**Affiliations:** 1 Missouri Institute of Mental Health, University of Missouri, St. Louis, MO, USA

## 

There is a growing body of evidence that screening, brief intervention, and referral to treatment (SBIRT) is effective in reducing drinking and substance use problems. Based on the outcomes from MOSBIRT, Missouri’s Substance Abuse and Mental Health Services Administration (SAMHSA)-funded SBIRT program, individuals receiving brief interventions show reductions in risky use, along with improvements in employment, housing, legal involvement and physical and mental health. As numerous studies indicate a cost savings of $3.81 to $5.60 for each dollar invested in screening for risky use, the most promising approach to sustaining SBIRT services in Missouri after SAMHSA support expires is to fund the state Medicaid codes already on the state’s fee schedule, thereby allowing for reimbursement of SBI services. To that end, an analysis of the Medicaid fee schedules of the 50 states and DC was conducted to determine which states currently have SBI Medicaid codes H0049 and H0050, as well as the American Medical Association’s Common Procedural Terminology (CPT) codes 99408 and 99409, on their schedules. The analysis indicated that 22 states have either the Medicaid or CPT codes with associated billing amounts on their Medicaid fee schedules. The average billing rate for screening is about $25 per unit, with rates of around $40 per unit for brief interventions. This information was presented to the Missouri governor’s budget office and resulted in rate setting and coverage of SBI services at federally qualified health centers and community mental health centers across the state. A rationale for supporting SBI services nationally is provided, along with recommendations for petitioning state budget offices to fund the Medicaid codes based on lessons learned from the MOSBIRT project.

